# The Composition, Antioxidant and Antibacterial Activities of Cold-Pressed and Distilled Essential Oils of* Citrus paradisi* and* Citrus grandis* (L.) Osbeck

**DOI:** 10.1155/2015/804091

**Published:** 2015-11-22

**Authors:** Ming-Chiu Ou, Yi-Hsin Liu, Yung-Wei Sun, Chin-Feng Chan

**Affiliations:** ^1^Department of Applied Cosmetology, Hungkuang University, No. 1018, Sec. 6, Taiwan Boulevard, Shalu District, Taichung 43302, Taiwan; ^2^Taiwan Seed Improvement and Propagation Station, COA, Taichung 42642, Taiwan

## Abstract

The chemical composition and functional activities of cold-pressed and water distilled peel essential oils of* Citrus paradisi* (*C. paradisi*) and* Citrus grandis* (L.) Osbeck (*C. grandis*) were investigated in present study. Yields of cold-pressed oils were much higher than those of distilled oils. Limonene was the primary ingredient of essential oils of* C. paradisi* (cold 92.83%; distilled 96.06%) and* C. grandis* (cold 32.63%; distilled 55.74%). In addition,* C. grandis* oils obtained were rich in oxygenated or nitrogenated compounds which may be involved in reducing cardiovascular diseases or enhancing sleep effectiveness. The order of free radical scavenging activities of 4 citrus oils was distilled* C. paradisi* oil > cold-pressed* C. paradisi* oil > distilled* C. grandis* oil > cold-pressed* C. grandis* oil. Cold-pressed* C. grandis* oil exhibited the lowest activity in all antioxidative assays. The order of antimicrobial activities of 4 citrus oils was distilled* C. grandis* oil, cold-pressed* C. paradisi* oil > distilled* C. paradisi* oil > cold-pressed* C. paradisi* oil. Surprisingly, distilled* C. grandis* oil exhibited better antimicrobial activities than distilled* C. paradisi* oil, especially against* Escherichia coli* and* Salmonella enterica* subsp. The results also indicated that the antimicrobial activities of essential oils may not relate to their antioxidative activities.

## 1. Introduction

Citrus essential oils have become increasingly important in food, alternative medicines, and cosmetic industries owing to their high yields, aromas, flavors, and many biological activities [1]. Citrus peel essential oils have been demonstrated with a broad spectrum of activities on reducing anxiety, depression [2], and cancer pain and on increasing sedative activities [3, 4]. In recent years, citrus essential oils have attracted more attention as antifungal agents owing to their antimicrobial properties [5]. Therefore, demand for citrus peel oils is increased in food, drugs, and cosmetic industries [6].

Essential oils can be obtained from plants by cold-pressed, water distilled, and solvent extraction and so forth. Cold-pressed and water distilled extractions are the most traditional and commonly used methods for citrus oils from plant material. The main constituents found in essential oils may include alkenes, acids, alcohols, aldehydes, esters, ketones, phenols, and nitrogenated compounds [7]. The chemical composition and biological efficacy can be dramatically different in oils extracted from different varieties of citrus or from identical citrus materials using different extraction methods [8].

The present study analyzed the composition of cold-pressed and water distilled peel essential oils of* Citrus grandis* (L.) Osbeck (*C. grandis*) and* Citrus paradisi* (*C. paradisi*) in Taiwan and evaluated the antioxidative and antimicrobial activities of 4 essential oils. In addition, their antibacterial activities against Gram-negative bacteria and Gram-positive bacteria were investigated.

## 2. Material and Methods

### 2.1. Collection of Plant Material

*C. paradisi*, Star Ruby grapefruit was from Kukeng, Yunlin County, in southern Taiwan.* C. paradisi* was harvested at late August 2011 and collected at early September 2011.* C. grandis* was cultivated in Matou, Tainan city, in Taiwan and collected and provided by Chang-Ching Fruits & Vegetables Logistics and Distribution, Kaohsiung, Taiwan, at October 2011. Both* Citrus paradisi* and* Citrus grandis *(L.) Osbeck were identified by Dr. Yung-Wei Sun, Taiwan Seed Improvement and Propagation Station, COA, Taichung City, Taiwan.

### 2.2. Chemicals

1,1-Diphenyl-2-picrylhydrazyl (DPPH), 2,2′-azinobis-3-ethylbenzothiazoline-6-sulfonate (ABTS), phosphate buffered saline, butylated hydroxytoluene (BHT), ferrozine, EDTA-2Na, and methyl alcohol were purchased from Sigma-Aldrich Co. (St. Louis, MO). Trichloroacetic acid was purchased from Acros Co. Thermo Fisher Scientific (Geel, Belgium). CuSO_4_, K_3_Fe(SCN)_6_, FeSO_4_·7H_2_O, and FeCl_3_ were purchased from Showa Chemical Industry Company Ltd. (Tokyo, Japan). Nutrient Broth, Nutrient Agar, Tryptic Soy Broth, and Tryptic Soy Agar were purchased from Becton, Dickinson and Company (Sparks, MD).

### 2.3. Microbial Strains

The essential oils were individually tested against a panel of microorganisms including Gram-negative bacteria,* Escherichia coli *(ATCC 8739),* Salmonella enterica* subsp. (ATCC 11511), and* Pseudomonas aeruginosa *(ATCC 15522), and Gram-positive bacteria,* Staphylococcus aureus *(ATCC 6538) and* Streptococcus faecalis *(ATCC 29212). The microbial strains were from Bioresource Collection and Research Centre, Food Industry Research and Development Institute, Hsinchu, Taiwan.

### 2.4. Cold-Pressed Extraction of the Essential Oils

In brief, fresh* C. grandis* and* C. paradisi* were washed and then peeled. The peels were dried, and then the white inner membranes were removed. Approximately two kilograms of* C. grandis* or* C. paradisi* peels was pressed at room temperature by sugarcane juice presser (Taiwan), respectively. The juice was collected and centrifuged at 6,000 rpm, for 30 min. The upper layer was further subjected to anhydrous sodium sulfate to remove excess water.

### 2.5. Water-Distilled Extraction of the Essential Oils

The fresh air dried* C. grandis* peels (700–800 g) or* C. paradisi* peels (700–800 g) were subjected to water-distillation boiling (>100°C) for 3 h by using a Clevenger-type apparatus. The obtained essential oil was dried over anhydrous sodium sulfate and after filtration stored at 4°C until tested.

### 2.6. Gas Chromatography- (GC-) MS Analysis

The Hosni et al. modified method was used to determine the chemical composition of citrus essential oils [9]. For the identification of volatile components, each sample was analyzed by GC-MS QP 2010 (Shimadzu, Seisakusho, Japan) equipped with BP-X5 capillary column (30 m·0.25 mm; coating thickness 0.25 *μ*m). Analytical conditions were injector temperature, 250°C; carrier gas helium at 1 mL/min; injection mode, split ratio, 1 : 100; volume injected, 1 *μ*L of a solution in methanol of the oil; and oven temperature programmed from 70°C to 280°C at 10°C/min and maintained for 5 minutes at 280°C. The MS scan conditions used included a transfer line temperature of 250°C, an interface temperature of 250°C, an ion source temperature of 200°C, ionization technique, electronic impact (EI) at 70 eV, an acquisition range of 30–300* m*/*z*, and a scan rate of 1 amu/s. Identification of the constituents was based on comparison of the retention times and on computer matching against NIST 97 MS Data library. When possible reference compounds were cochromatographed to confirm GC retention times.

### 2.7. DPPH Scavenging Effect

The scavenging activity of oils on 1,1-diphenyl-2-picrylhydrazyl radical (DPPH) was determined using the method described by Liao et al. [10]. Fifty microliters of different concentrations (10 mg/mL, 20 mg/mL, and 40 mg/mL) of essential oils was mixed with 150 *μ*L of freshly prepared 0.5 mM DPPH in ethanol and ascorbic acid was used as positive control. The mixture was kept in the dark for 30 min. DPPH absorbance was then measured at 517 nm, using ELISA reader (Tecan, Austria). Percent of scavenging effect was calculated using the following equation:

(1) $$\begin{array}{c} \text{Scavenging effect }\left(\;\%\;\right)=1-\left(\;\frac{A_{\text{Sample 517 nm}}}{A_{\text{Control 517 nm}}}\;\right)\times 100. \end{array}$$

Each test was carried out in triplicate.

The DPPH assay is commonly used because it is operationally simple and rapid. The other advantage is that a large number of samples could be measured simultaneously by using microplates and gives accurate and repeatable results. There are some limitations of DPPH assay. DPPH is a stable nitrogen radical and has no similarity to the highly reactive and transient radicals such as peroxyl radicals in living organism [11]. Therefore, antioxidants that react quickly with peroxyl radicals may react very slowly to the DPPH radical. Time response curve of interaction between DPPH radical and different ratios of antioxidant is not linear [12]. Moreover, interpretation of the results is complicated if the antioxidants, for example, carotenoids, have absorbance spectra that overlap with DPPH at 517 nm [13].

### 2.8. ABTS Scavenging Effect

The Wootton-Beard [14] modified method was used to determine scavenging activity of ABTS radical cation. ABTS^∙+^ was generated by mixing a 7 mM aqueous solution of ABTS with 2.45 mM potassium persulfate (final concentration) followed by storage in the dark at room temperature for 10 h before use. The reaction mixture was diluted with ethanol to give an absorbance of 0.7 ± 0.01 units at 734 nm using the ELISA reader (Tecan Sunrise, Tecan Austria GmbH). The 20 *μ*L different concentrations (10 mg/mL, 20 mg/mL, and 40 mg/mL) of essential oils were reacted with 180 *μ*L fresh ABTS^∙+^ solution, and absorbance was then measured 3 min after initial mixing. Each test was carried out in triplicate.

ABTS can be solubilized both in aqueous and in organic media and permits study over a wide pH range. However, there are also some limitations of ABTS assay. The reaction between ABTS and antioxidant may take a long time to reach an end point. The other limitation is that the reaction products may have more contribution to scavenging of ABTS than parent compound [11].

### 2.9. Iron Chelating Activity

The iron chelating capacity of the sample was determined, using the method proposed by Dinis et al. [15]. 25 *μ*L different concentrations of essential oils were mixed with 175 *μ*L of methanol, 25 *μ*L of 400 *μ*M FeCl_2_·4H_2_O, and 25 *μ*L of 2 mM ferrozine. The mixture was allowed to stand for 10 min, and the absorbance was then measured at 562 nm, using ELISA reader (Tecan, Austria). EDTA was used as positive control. Each test was carried out in triplicate.

The transition metal ion ferrous can promote formation of free radicals by gain or loss of electrons. Therefore, to chelate the transition metal ions with chelating agents also can reduce the formation of reactive oxygen species [12]. These chelating agents that can slow the rate of oxidation but do not convert free radicals to stable products were usually used in food and cosmetic industries as secondary antioxidants.

### 2.10. Reducing Power

The Singh and Rajini [16] method was used to determine the reducing power of the extracts. A total of 100 *μ*L of oils at different concentrations (1 mg/mL, 2 mg/mL, 4 mg/mL, 10 mg/mL, and 20 mg/mL) was mixed with 100 *μ*L of 0.2 M phosphate buffer and pH 6.6 and 100 *μ*L of 1% (w/v) K_3_Fe(CN)_6_. Ascorbic acid was used as positive control. The mixture was incubated at 50°C for 20 min in a water bath. Ten percent (10% w/v) trichloroacetic acid (100 *μ*L) was added and the resulting mixture was centrifuged (at 3000 rpm) for 10 min. One hundred microliters of the supernatant was combined with 100 *μ*L of distilled water and 20 *μ*L of 0.1% (w/v) FeCl_3_ solution. The absorbance was measured at 700 nm, using the V630 UV-Vis Spectrophotometer (JASCO Co., Ltd., Japan). Interpolation from the linear regression analysis of absorbance was 0.5 for EC_50_ of reducing power [17]. Each test was carried out in triplicate.

The advantage of the reducing power assay is its simplicity and quick and inexpensive and available instruments. The limitation of the reducing power assay is that any reagent with a redox potential lower than 0.77 V may drive ferrous iron formation, even though it may not behave as an antioxidant* in vivo* [13]. Reducing power is related to the extent of conjugation in phenols and the number of hydroxyl constituents. Protein and thiol antioxidants cannot be measured by the reducing power assay [12, 13].

### 2.11. Microbial Strains Culture

*Escherichia coli*,* Pseudomonas aeruginosa*,* Salmonella enterica* subsp., and* Staphylococcus aureus* were cultured in Nutrient Broth.* Streptococci aureus* was cultured in Tryptic Soy Broth. All strains were cultured overnight in a rotary shaker at 37°C. The cultures were centrifuged at 10,000 rpm for 5 min. The pellets were resuspended in double distilled water and cell density was standardized with a spectrometer (*A*_610_ nm) [18].

### 2.12. Agar Disc Diffusion Method

Antimicrobial activity of the essential oils was evaluated in agreement with the method of Kiraz et al. [19]. For the determination of antimicrobial activity, bacterial cultures were adjusted to 10^8^ CFU/mL. Then, 0.1 mL amounts of each culture were pipetted into separate sterile Petri dishes, and 9.9 mL amounts of molten Tryptic Soy Agar (45°C) were added. Once set, wells of 5 mm in diameter were formed in each agar plate using a Pharmacia gel punch (Uppsala, Sweden). The plates were then left undisturbed to allow diffusion of the 100 *μ*L of dilution samples into the agar and were incubated in the dark at 37°C for 24 h. The zones of growth inhibition were then measured using Vernier calipers. Dimethyl sulfoxide was served as a vehicle control and streptomycin was served as a positive antibacterial control. For each extract 3 replicate trials were conducted against each organism. A diameter of inhibition zone (IZ) less than 6 mm was indicating no antimicrobial effect, with 6 mm < IZ < 9 mm indicating moderate antimicrobial effect, 10 mm < IZ < 14 mm indicating strong antimicrobial effect, and IZ > 15 mm indicating very strong antimicrobial effect [20, 21].

### 2.13. Statistics

Three samples were prepared for each assay. The results were expressed as means and standard deviation. Data analysis included one-way ANOVA, followed by Duncan's multiple range test (*p* < 0.05) and a correlation test using the SigmaStat 3.5 program.

## 3. Results

### 3.1. Yield and Physical Analysis of Citrus Peel Essential Oils

The oils of cold-pressed* C. paradisi* and* C. grandis* were light orange and light yellow, respectively. The oils of water distilled* C. paradisi* and* C. grandis* oils were transparent. The yields of essential oils from cold-pressed* C. paradisi* and* C. grandis* were high, approximately 16.41% and 14.25%, respectively. However, the yields of essential oils from distilled* C. paradisi* and* C. grandis *were only 0.37% and 0.29%, respectively (Table 1). The yields of* C. grandis* essential oils by cold-pressed extraction (14.25%–16.41%) are significantly higher than the values reported by Song et al. [(0.15%–0.27%) [22]], Blanco Tirado et al. [(0.60%–0.79%) [23]], and Lota et al. [(0.60%) [24]]. The yields of water-distilled essential oils ranged from 0.29% to 0.37% which were slightly less than the results (1.06% to 4.62%) of Hosni et al. [9] but higher than the results (0.17%–0.21%) of Blanco Tirado et al. [23]. Therefore, yields of oils could be different because of the different extraction conditions, origins, seasons, and environmental factors.

### 3.2. Composition of Oils Analyzed by GC-MS

The main ingredients of* C. grandis* and* C. paradisi* essential oil analyzed by GC-MS were terpenes, including *α*-pinene and limonene, in which the retention time was 3.62 min and 4.78 min, respectively (Table 1). Limonene contents of* C. paradisi* oils were cold-pressed 92.83% and distilled 96.06% and* C. grandis* oils were cold-pressed 32.63% and distilled 55.74%, respectively (Table 1).

The chemical composition between cold-pressed and distilled oils was different. Distilled* C. grandis *oil contained *β*-pinene (14.74%), linalool (6.23%), *β*-citral (4.13%), and *α*-citral (4.6%) as shown in Table 1. Cold-pressed and distilled* C. paradisi* oils primary contained terpene (limonene and *α*-pinene, thujene, and *β*-myrcene), the sesquiterpene vinyl (*β*-caryophyllene), and aldehyde (decyl aldehyde). Cold-pressed* C. grandis* contains terpene (*β*-pinene and limonene) and fatty acid (oleic acid and palmitic acid), and ammonia and a high proportion of fatty acid amide derivatives oil (oleylamide, 20.38%). Distilled* C. grandis *contains a higher proportion of oxygen-containing compounds such as aldehydes (*α*-citral and *β*-citral), alcohol (linalool), and monoterpene (limonene and *α*-pinene and *β*-pinene) as shown in Table 1.

### 3.3. DPPH Measurement

*C. grandis *oils and cold-pressed* C. paradisi* oil displayed weak DPPH radicals scavenging capability. DPPH scavenging capacity of cold-pressed* C. paradisi* oil was less than 20% (Table 2). Distilled* C. paradisi* oil exhibited the potent DPPH scavenging capacity among 4 citrus oils; the EC_50_ value was more than 40 mg/mL. This is consistent with previous studies where 34 kinds of 10 mg/mL citrus oils obtained from Japan and Korea and Italy exhibited weak DPPH radical scavenging effect ranging from 12% to 17.7% [25].

### 3.4. ABTS Measurement

ABTS clearance rate of 40 mg/mL distillation* C. paradisi* oil was 66.14% and EC_50_ value was 25.7 mg/mL; however the clearance rate for 40 mg/mL cold-pressed* C. paradisi* oil was only 15.94% (Table 2). ABTS clearance rates of 40 mg/mL distilled-pressed and cold-pressed* C. grandis *oils were only 9.06% and 6.00%, respectively (Table 2). Therefore, ABTS free radical scavenging ability of 4 citrus oils was distilled* C. paradisi* oil > cold-pressed* C. paradisi* oil > distilled* C. grandis* oil > cold-pressed* C. grandis* oil.

### 3.5. Iron Chelating Activity

**Figure 1:**
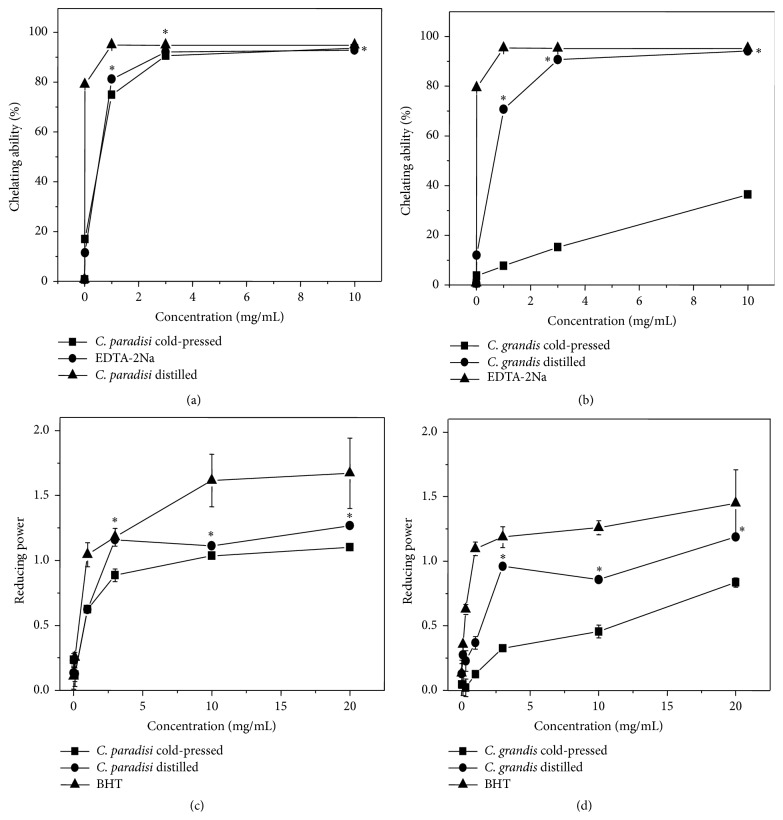
Iron chelating activity and reducing power of essential oils of* C. paradisi* and* C. grandis*. (a) Iron chelating activity of cold-pressed and distillated essential oils of* C. paradisi.* (b) Iron chelating activity of cold-pressed and distilled essential oils of* C. grandis*; EDTA-2Na was used as positive control. (c) Reducing power of cold-pressed and distilled essential oils of* C. paradisi.* (d) Reducing power of cold-pressed and distillated essential oils of* C. grandis*; BHT was used as a positive control. Values are means ± SD (*n* = 3). An asterisk indicates a significant difference to the control (^*∗*^ *p* < 0.05).

Regardless of being cold-pressed or distilled,* C. paradisi* oils had good ferrous ion chelating ability. At 1 mg/mL, cold-pressed and distilled* C. paradisi* oils exhibited 71% and 78% ferrous chelating ability, respectively (Figure 1(a)). The EC_50_ value for cold-pressed* C. paradisi* oil was 0.5 mg/mL and for distilled* C. paradisi* oil was 0.6 mg/mL (Figure 1(a)). EDTA-2Na was used as a positive control for ferrous chelating ability.

The ferrous chelating ability of cold-pressed* C. grandis* oil exhibited the lowest activity among 4 citrus oils; the EC_50_ value was 11.2 mg/mL. Distilled* C. grandis* oil also exerted potent ferrous chelating ability; the EC_50_ value was 1.4 mg/mL (Figure 1(b)).

### 3.6. Reducing Power

The reducing powder of 1 mg/mL* C. paradisi* oil obtained by cold-pressed method was 63% capabilities of 1% BHT. For the distilled* C. paradisi* oil, 1 mg/mL of oil exhibited approximately 78% capabilities of 1% BHT which was used as a positive control (Figure 1(c)). The result demonstrated that distilled* C. paradisi* oil was much potent than cold-pressed oils in reducing power (Figure 1(c), ^*∗*^ *p* < 0.05). The reducing power of cold-pressed and distilled* C. grandis* oils was not as potent as in the* C. paradisi* oils (Figure 1(d)).

### 3.7. Agar Disc Diffusion Measurement

Results showed that 10 mg/mL* C. paradisi* and* C. grandis* oils exhibited moderate inhibitory effects against Gram-positive bacteria but had no effect against Gram-negative bacteria except 10 mg/mL distilled* C. grandis* oil which exhibited moderate effect (IZ 9.3 mm) against* E. coli* (Table 3). Twenty milligram per milliliter* C. paradisi* and* C. grandis* oils obtained by cold-pressed or distilled method only had a moderate inhibitory effect against* P. aeruginosa* (maximum IZ 7.9 mm) (Table 3). Twenty milligram per milliliter cold-pressed* C. paradisi* oil and distilled* C. grandis* oil exhibited very strong inhibitory effects against* S. enterica* subsp. (IZ 20.6 mm and 21.6 mm, resp.) even stronger than effect of streptomycin (IZ 17.3 mm). Twenty milligram per milliliter cold-pressed* C. paradisi* oil and distilled* C. grandis* oil also exhibited strong inhibitory effects against* E. coli* (IZ 12.9 mm and 14.6 mm, resp.). All 20 mg/mL oils except cold-pressed* C. grandis* oil (IZ 8.9 mm) exhibited strong inhibitory effects against* S. aureus*. All two-percent oils except cold-pressed* C. grandis* oil (IZ 8.9 mm) exhibited very strong inhibitory effects against* S. faecalis *(IZ 17.3 mm) which were even better than effect of streptomycin (IZ 10.9 mm). Streptomycin (10 *µ*g/mL) was used as a positive control for inhibition of bacterial growth in present study.

In general, the order of antimicrobial activities of 4 citrus oils was distilled* C. grandis* oil, cold-pressed* C. paradisi* oil > distilled* C. paradisi* oil > cold-pressed* C. paradisi* oil.

## 4. Discussion

Previous reports showed that the main components of different varieties of cold-pressed citrus peel oils were limonene (62.5%–95.7%), *γ*-terpinene (0.1%–23.3%), *α*-pinene (0.1%–2.5%), and myrcene (1.7%–2.0%) [26] and that was consistent with the main ingredients of* C. paradisi* oil in present study, limonene (91.83%), *β*-myrcene (3.06%), and *α*-pinene (0.85%) (Table 1). However, cold-pressed* C. grandis* peel oils obtained in present study had high content of oxygenated compounds such as fatty acids and nitrogenated compounds which were usually obtained in seed of citrus rather than in the citrus peel [27]. The fatty acid obtained from citrus seeds included saturated and unsaturated fatty acid, such as linoleic acid, oleic acid, stearic acid, and epoxyeicosatrienoic acids [27]. In present study, cold-pressed peel essential oils contained oleic acid, 6-octadecenoic acid, palmitic acid, and stearic acid (Table 1). Unsaturated fatty acids were able to reduce the low density lipoprotein (LDL) and prevent arteriosclerosis [28]. Cold-pressed* C. grandis* oils also contained nitrogen-containing derivatives, oleylamide. In fact, oleylamide also is an endogenous substance that can combine with cannabinoid receptors (cannabinoid 1, CB1) and reduce pain or increase sensation of pleasure. In addition, oleylamide can encourage people to fall asleep by promoting the reaction of gamma-amino butyric acid (GABA) receptor [29]. Therefore, cold-pressed* C. grandis* oil may encourage pleasant mood and enhance sleep effectiveness.

The main ingredients of water distilled* C. grandis* (from Korea) oil in Baik et al.'s report [30] were limonene (68.08%), *β*-myrcene (22.65%), and *γ*-terpinene (1.63%) and the main ingredients of this study were limonene (55.74%), *β*-pinene (14.74%), and linalool (6.23%). The second and the third main ingredients of two water distilled* C. grandis* essential oils were different. The result indicated that main ingredients of essential oils of same plant variety cultivated in different regions may still have considerable differences. The main ingredients of water distilled* C. paradisi* oil in El Kamali et al.'s report [31] were limonene (74.45%), *β*-myrcene (12.85%), and *α*-pinene (3.74%) that were consistent with the main ingredients of water distilled* C. paradisi* oil in present study which were limonene (96.06%), *β*-myrcene (2.06%), and *α*-pinene (0.52%).

It has been suggested that limonene, *α*-terpinolene, *β*-caryophyllene, *β*-pinene, and myrcene and geraniol of citrus oils had high antioxidative activity [5, 32]. The free radical scavenging activity of* C. paradisi* and* C. grandis* oils was in the same order as the content of limonene of 4 citrus oils (Tables 1 and 2). The results indicated that limonene may play a pivotal role in antioxidative activities of* C. paradisi* and* C. grandis* oils.

The composition of limonene, *α*-pinene, *γ*-terpinene, geraniol, thymol, *β*-pinene, sabinene, *β*-myrcene, *β*-citral (Neral), and *α*-terpineol of* C. paradisi* or* C. grandis* oils (Table 1) has been demonstrated with different sensitivity against various microbes [5, 33, 34]. Antimicrobial activity also is dependent on volatility stability and hydrophobicity of compounds. Limonene has high volatility, easy oxidation, and low solubility in water which indicate that it cannot be absorbed by agar. Therefore, high contents of limonene may not result in high antimicrobial activity [35]. This is consistent with the results of distilled* C. paradisi* oil which had the highest content of limonene but did not exhibit the best antimicrobial activity among 4 citrus oils (Tables 1 and 3). Previous reports have also demonstrated that the most active antimicrobial ingredients of essential oils are aldehyde, phenol, and alcohol followed by ketone, ether, and hydrocarbon, especially hydrocarbon as a relative weak constituent [5, 35]. That maybe is the reason why distilled* C. grandis* oil which had lower content of limonene but higher contents of potent antimicrobial composition, *α*-citral, *β*-citral, *γ*-terpinene, and *α*-terpineol (Table 1), still exhibited the best antimicrobial activities among 4 citrus oils (Table 3).

In general, the results showed that distilled* C. paradisi* oils had the best antioxidative activity; cold-pressed* C. paradisi* oil exhibited potent antimicrobial activity and was obtained with high-yield extraction; distilled* C. grandis* oil exhibited the best antimicrobial activity; cold-pressed* C. grandis* oil exhibited the lowest radical scavenging and antimicrobial activities but may have effects on enhancing pleasant mood and sleep effectiveness among 4 citrus oils. The chemical composition and bioactivities were discussed in the present study in an attempt to provide new information for the utilization of citrus peel essential oils in the future.

## Figures and Tables

**Table 1:** The composition of essential oils of *C. paradisi* and *C. grandis* by GC-MS.

Retention time	Composition	*C. paradisi*		*C. grandis*	
		Cold-pressed	Distilled	Cold-pressed	Distilled
3.62	*α*-Pinene	0.85	0.52	0.33	2.31
4.07	Thujene	0.8	0.39		
4.09	Sabinene			0.29	1.34
4.17	*β*-Myrcene	3.06	2.06		
4.19	*β*-Pinene			2.35	14.74
4.51	*α*-Phellandrene				0.13
4.78	Limonene	91.83	96.06	32.63	55.74
4.90	*β*-Ocimene	0.15		0.14	
5.16	*γ*-Terpinene		0.18		0.64
5.34	Linalool				6.23
5.56	Dihydrocarveol				0.57
6.43	Citronellal				0.17
6.96	4-Terpinenol		0.21		0.55
7.14	Isopulegol	0.38			
7.16	Decyl aldehyde		0.39		
7.17	*α*-Terpineol				1.98
7.24	Geraniol				1.58
7.48	Carveol		0.19		
7.70	*β*-Citral				4.13
7.79	Lemonol				0.75
7.90	Carvone				0.48
8.10	*α*-Citral (Neral)				4.6
9.04	1-Pentadecyne	0.2			
9.47	Neryl acetate				0.31
9.59	*α*-Copaene	0.2			
9.76	*β*-Elemene				0.12
10.23	*β*-Caryophyllene	0.43			
10.38	*β*-Cubebene				0.13
10.41	*β*-farnesene	0.23			
11.02	Germacrene D	0.35		0.61	2.71
11.11	Farnesene				0.12
11.24	*γ*-Elemene				0.37
11.41	*β*-Cadinene	0.28			
13.46	*β*-Sinensal	0.34			
13.67	Farnesol				0.3
13.94	Myristic acid			3.21	
14.95	Nootkatone	0.19			
15.10	Pentadecanoic acid			1.05	
16.18	Palmitic acid			13.67	
17.37	Stearyl alcohol			0.62	
17.90	Oleic acid			6.57	
18.10	Stearic acid			4.28	
18.37	Hexadecanamide			2.14	
18.45	Octadecyl acetate			0.33	
19.97	Oleylamide			20.38	
22.45	Allyl stearate			0.6	
23.80	Erucamide			2.01	
23.94	Spinacene			3.04	

Terpenes		98.52	99.21	39.39	78.35
Oxygenated compounds		0.77	0.79	30.46	21.65
Others (nitrogenated or sulfated compounds)				23.68	

**Table 2:** DPPH and ABTS scavenging activity of cold-pressed and distilled essential oils of *C. paradisi* and *C. grandis*.

Oil concentration	*C. paradisi*		*C. grandis*	
	Cold-pressed	Distilled	Cold-pressed	Distilled
DPPH scavenging activity				
10 mg/mL	0.88 ± 0.01^b^	14.87 ± 0.01^*∗*a^	0	0
20 mg/mL	1.12 ± 0.00^b^	32.13 ± 0.00^*∗*a^	0	0
40 mg/mL	7.75 ± 0.01^b^	51.24 ± 0.01^*∗*a^	0	2.32 ± 0.01^c^

ABTS scavenging activity				
10 mg/mL	7.21 ± 0.011^b^	27.42 ± 0.019^*∗*a^	3.11 ± 0.006^d^	3.70 ± 0.006^c^
20 mg/mL	10.47 ± 0.009^b^	48.36 ± 0.016^*∗*a^	3.66 ± 0.009^d^	4.95 ± 0.015^c^
40 mg/mL	15.94 ± 0.006^b^	66.14 ± 0.005^*∗*a^	6.00 ± 0.010^d^	9.06 ± 0.007^c^

Values are means ± SD (*n* = 3). An asterisk indicates a significant difference to the control (^*∗*^ *p* < 0.05). Means in a raw with different small letters are significantly different (^*∗*^ *p* < 0.05).

**Table 3:** Inhibition zones of cold-pressed and distilled essential oils of *C. paradisi* and *C. grandis*.

Species	Inhibition zone of essential oils (mm)								
	*C. paradisi*				*C. grandis*				Streptomycin
	Cold-pressed		Distilled		Cold-pressed		Distilled		
	10 mg/mL	20 mg/mL	10 mg/mL	20 mg/mL	10 mg/mL	20 mg/mL	10 mg/mL	20 mg/mL	10 *μ*g/mL
*P. aeruginosa*	—^#^	6.9 ± 0.6	—	6.3 ± 0.6	—	7.9 ± 0.6	—	6.3 ± 0.6	18.4 ± 1.3
*S. enterica *subsp.	—	21.6 ± 1.0	—	10.7 ± 1.5	—	6.6 ± 1.0	—	20.6 ± 1.0	17.3 ± 1.2
*E. coli *	—	12.9 ± 1.5	—	10.6 ± 0.0	—	7.6 ± 1.0	9.3 ± 0.6	14.6 ± 1.0	17.6 ± 0.9
*S. aureus*	7.3 ± 1.5	13.6 ± 1.0	8.9 ± 2.1	12.3 ± 0.6	9.3 ± 1.2	8.9 ± 1.5	7.6 ± 1.0	13.6 ± 1.0	20.7 ± 0.9
*S. faecalis*	10.6 ± 1.0	17.3 ± 5.0	9.3 ± 0.6	17.3 ± 2.3	6.0 ± 0.6	8.9 ± 0.6	8.6 ± 2.6	17.3 ± 1.5	10.9 ± 1.2

^#^Degree of inhibition: —: no inhibition zone (≦6 mm). Moderate inhibition zone (6–9 mm). Strong inhibition zone (10–14 mm). Very strong inhibition zone (>15 mm).
